# Empowering minds: how self-efficacy, self-esteem, and social support drive digital mental health engagement

**DOI:** 10.3389/fpubh.2025.1565327

**Published:** 2025-04-25

**Authors:** Jack Ng Kok Wah

**Affiliations:** Faculty of Management, Multimedia University, Cyberjaya, Selangor, Malaysia

**Keywords:** e-health technologies, self-efficacy, perceived social support, self-esteem, digital mental health engagement

## Abstract

**Introduction:**

Despite the rapid growth of research in digital health, there is a significant gap in understanding how psychological factors such as self-efficacy, self-esteem, and perceived social support collectively influence digital mental health engagement, particularly within the Malaysian context. While previous studies have explored these constructs individually, few have examined their integrated effects on user engagement with e-health platforms. The study aims to fill that gap by exploring the direct relationships between self-efficacy, self-esteem, and perceived social support with digital mental health engagement, while also analyzing the mediating role of perceived social support. The novelty of the research lies in integration of these psychological constructs into a unified conceptual framework to provide a more comprehensive understanding of digital mental health engagement.

**Methods:**

Using a quantitative, cross-sectional design, the study surveyed 400 active Malaysian e-health users through a self-administered questionnaire. The survey used validated scales to measure self-efficacy, self-esteem, perceived social support, and engagement with digital mental health platforms. Structural Equation Modeling (SEM) was employed to test five hypotheses regarding direct and mediated relationships.

**Results:**

Demographic analysis revealed that many participants were female (71.3%), aged between 25 and 45 years (76.6%), and from higher income brackets (RM5,001–RM20,000). WhatsApp (400 users) was found to be the most popular tool, followed by Facebook (387 users) and Instagram (371 users), highlighting the importance of these platforms in connectivity and information sharing. The study found that perceived social support had the strongest direct effect on digital mental health engagement (β = 0.523), followed by self-esteem (β = 0.384) and self-efficacy (β = 0.236). Additionally, perceived social support significantly mediated the impact of both self-efficacy and self-esteem on engagement.

**Discussion:**

These findings underscore the importance of fostering supportive digital environments to enhance users’ confidence and self-worth. However, the cross-sectional nature of the study limits causal interpretations, and the localized sample restricts generalizability. Future research should incorporate longitudinal methods and explore cultural differences. Overall, the study contributes to the development of effective digital mental health engagement strategies in Malaysia and beyond.

## Introduction

In the digital age, e-health platforms have become transformative tools in mental health care, providing individuals with access to resources, support networks, and tailored interventions. Digital mental health encompasses mobile apps, online platforms, telehealth, and eHealth (30) tools used to assess, monitor, and treat mental health conditions, promoting psychological well-being and self-management (1). The evolving field integrates psychological theories and digital innovation to enhance access to mental health services, particularly for populations facing barriers to traditional care. Mental health professionals stress the importance of ethical, culturally sensitive digital interventions, especially in multicultural and low-resource settings (2, 3).

### Background

The increasing prevalence of digital mental health challenges has amplified the demand for innovative, accessible solutions. e-health platforms, utilizing technology for psychological support, counseling, and monitoring services, have transformed mental well-being management. Key factors influencing the success of these platforms include self-efficacy, self-esteem, and perceived social support (29). These psychological constructs determine how individuals engage with e-health tools and their subsequent digital mental health engagement. As digital mental health engagement becomes pivotal in contemporary healthcare, understanding these psychological determinants is crucial. Self-efficacy, self-esteem, and perceived social support significantly impact motivation, confidence, and emotional well-being in navigating digital health resources (4, 5).

### Theoretical framework

The study’s theoretical framework explores the interplay between self-efficacy, self-esteem, perceived social support, and e-health engagement within the context of digital mental health. Building on Bandura’s (4) self-efficacy theory, which emphasizes the role of confidence in performing health-related tasks (5), the framework positions self-efficacy and self-esteem as independent variables, perceived social support as a mediator, and e-health engagement as the dependent variable. Prior studies support the mediating role of social support and self-esteem in digital mental health contexts, such as Alquaiz et al. (6) on violence against women, and broader evidence from Cohen (7) and Uchino (8). Appendix A highlights comparative findings across various studies and settings, underscoring the relevance of these psychological factors.

The significance of the framework is further demonstrated through research on digital mental health tools and interventions. e-health platforms have proven effective in managing chronic conditions and mental health challenges (3, 9). Digital environments, including social media, are increasingly shaping mental health outcomes, influencing body image, depression, and anxiety (10, 11). Specific case studies, such as autistic masking (12) and cancer survivorship (13), reinforce the applicability of the framework. The findings show that self-efficacy enhances engagement (14), but the sustained use of digital tools relies heavily on perceived social support, aligning with Jiang et al. (15).

Moreover, the framework integrates Cohen’s (7) social support model, which posits that support systems buffer stress and improve well-being. The results confirm that social support boosts both self-efficacy and self-esteem, thereby increasing engagement in e-health (16, 17). However, unlike traditional models, the study emphasizes the role of digital communities in providing support (13). Self-esteem also predicts engagement (18), and the findings expand on Sociometer Theory (19), showing its dependence on digital social validation (11). Ultimately, the study underscores the importance of AI-driven and personalized digital mental health interventions to foster meaningful engagement (20).

### Key issues and gaps

e-health platforms are revolutionizing digital mental health care, yet several critical gaps remain. One key issue is the limited focus on self-efficacy, which is vital for health behavior change (21). While users with higher self-efficacy are generally more capable of navigating digital tools, research rarely explores how the trait helps overcome technical barriers or sustain long-term engagement. The role of app feedback in enhancing user confidence is also unclear. Similarly, self-esteem though closely linked to mental well-being (22) has not been thoroughly examined in relation to e-health engagement. Those with high self-esteem may be more inclined to utilize digital mental health resources, while those with low self-esteem might withdraw, potentially worsening their psychological state.

Another significant gap involves the underexplored role of perceived social support. While it is recognized for enhancing mental health resilience (16), how it interacts with self-efficacy and self-esteem in digital settings remains poorly understood. Although many platforms incorporate peer networks and professional assistance, the actual impact of perceived support on user engagement and mental health outcomes is still uncertain. Compounding methodological inconsistency, studies often use varying definitions of engagement, such as login frequency or time spent, making it difficult to compare results (3, 23). Furthermore, the lack of longitudinal data limits our understanding of sustained engagement and long-term effects. Most studies rely on cross-sectional designs, which do not capture the evolving nature of self-efficacy or the enduring benefits of digital mental health interventions (24).

### Aims

The study aims to address gaps in understanding how psychological factors influence e-health engagement and digital mental health. It examines self-efficacy’s role in fostering active participation, self-esteem’s impact on engagement and well-being, and the mediating effect of perceived social support. Additionally, it explores demographic, cultural, and gender-specific factors that moderate e-health effectiveness. The research provides evidence-based recommendations for designing personalized interventions that enhance psychological resilience, improve user experience, and optimize digital mental health engagement across diverse populations.

### Hypothesis

**Figure 1:**
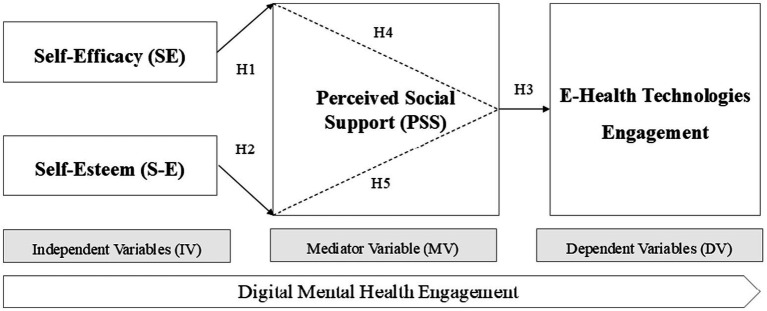
Conceptual model of the influence of self-efficacy, self-esteem, and perceived social support on digital mental health engagement.

To explore the relationships between psychological constructs and e-health engagement in promoting digital mental health, five hypotheses (Figure 1) were proposed. H1 suggests that self-efficacy, defined as an individual’s belief in their capabilities (4), significantly influences digital mental health engagement. According to Bandura’s theory, individuals with higher self-efficacy are more likely to engage in activities that promote their well-being, such as using e-health platforms. Therefore, H1 posits that greater self-efficacy will lead to more active participation in e-health engagement, which ultimately enhances mental health outcomes.

H2 extends this by proposing that self-esteem, which represents an individual’s sense of self-worth (25), also has a direct impact on digital mental health engagement. H2 suggests that individuals with higher self-esteem are more motivated to adopt behaviors that promote mental well-being, including engaging in e-health platforms. As self-esteem fosters confidence and a positive outlook, it may encourage individuals to seek and benefit from digital mental health resources.

H3 introduces perceived social support as another significant factor, stating that individuals who perceive higher levels of social support are more likely to engage with e-health platforms. Cohen (7) describes social support as emotional, informational, and instrumental assistance received from one’s social network. H3 posits that perceived social support enhances digital mental health engagement by providing individuals with the emotional reassurance needed to utilize e-health tools.

H4 proposes that perceived social support mediates the relationship between self-efficacy and e-health engagement. The hypothesis suggests that individuals who feel supported by their social networks are more likely to act on their self-efficacy beliefs and engage in e-health interventions.

Lastly, H5 suggests that perceived social support mediates the relationship between self-esteem and e-health engagement. The hypothesis argues that the positive influence of self-esteem on e-health engagement is contingent on the level of perceived social support, with individuals who feel supported being more likely to engage in digital mental health practices.

### Scope of the study

The study investigates how self-efficacy, self-esteem, and perceived social support influence e-health engagement and digital mental health. Using survey data from 400 users and Structural Equation Modeling, it emphasizes culturally varied contexts to explore psychological factors shaping user behavior and well-being in digital health platforms.

### Novelty contributions

The study enhances the understanding of digital mental health engagement by addressing critical gaps through five key contributions. It presents an integrated psychological framework that explores the interplay of self-efficacy, self-esteem, and perceived social support, offering a holistic view of their collective impact. Using Structural Equation Modeling (SEM), it delivers advanced analysis of complex relationships, surpassing traditional methods. The research emphasizes AI-driven, personalized e-health interventions tailored to individual psychological profiles. It also incorporates cultural and gender-specific considerations, promoting inclusivity. Finally, the study provides actionable insights for developers, practitioners, and policymakers to design more effective and accessible digital mental health solutions.

## Method

### Study design

The study adopts a quantitative, cross-sectional survey design to investigate the complex relationships between self-efficacy, self-esteem, perceived social support, and e-health engagement in the context of digital mental health. The approach allows for the examination of multiple psychological constructs and their interconnections at a single point in time, capturing current perceptions and behaviors. Utilizing validated measurement tools within a structured framework, the study offers a reliable and insightful snapshot of how these variables interact, contributing to a deeper understanding of the factors driving digital mental health engagement in today’s digital landscape.

### Participants and recruitment

A total of 400 active e-health and social media users in Malaysia were recruited using stratified random sampling to ensure diversity across age, gender, and cultural backgrounds, thus providing a representative sample of the population. Participants were eligible if they were at least 18 years old and had been actively engaged with e-health platforms or health-related social media networks for a minimum of six months. The criterion ensured that respondents possessed adequate experience and familiarity with digital health tools, allowing for meaningful insights into their psychological constructs and patterns of e-health engagement across different demographic segments.

### Data collection

Data collection was conducted using a self-administered questionnaire with a 5-point Likert scale (1 = strongly disagree to 5 = strongly agree) to capture participants’ perceptions and experiences. The questionnaire assessed key psychological constructs, including self-efficacy (4), self-esteem (25), and perceived social support (7), each selected for their relevance to digital mental health engagement. Demographic data such as age, gender, education level, and e-health platform usage frequency were also collected to provide context. The approach allowed for a comprehensive analysis of how psychological factors influence engagement with e-health platforms.

### Data analysis

The data were analyzed using Structural Equation Modeling (SEM) via SPSS and Smart-PLS software, chosen for its strength in assessing complex variable relationships while accounting for measurement error to improve model accuracy. The analysis involved a two-step approach: first, the Measurement Model was evaluated using Confirmatory Factor Analysis (CFA) to assess the reliability and validity of the constructs, with Cronbach’s alpha values exceeding 0.70, indicating strong internal consistency. Second, the Structural Model was examined through path analysis to test the proposed hypotheses and assess the mediating effect of perceived social support on the relationship between psychological constructs and e-health engagement.

### Ethical issues

Ethical approval for the study was obtained from the university’s ethics committee. Participants were fully informed about the study’s objectives, procedures, and potential risks, with voluntary consent obtained and the option to withdraw at any time without consequence. To prevent duplication or fraud, stratified random sampling was employed, ensuring diversity across age, gender, and cultural backgrounds, which helps create a representative sample and minimizes the risk of individuals submitting multiple responses under different identities.

The study also established specific eligibility criteria, requiring participants to be at least 18 years old and actively engaged with e-health platforms or health-related social media networks for at least six months. The criterion ensured that respondents had sufficient experience with digital health tools, enhancing the validity of their responses. Additionally, anonymity and confidentiality were strictly maintained, with personal identifiers removed. To minimize common method bias associated with self-reported data, the researcher carefully designed the questionnaire, utilizing clear and concise questions and counterbalancing question order to reduce response biases.

## Results

### Demographic insights

The demographic profile of the 400 e-health users who participated in the study plays a crucial role in assessing the representativeness and generalizability of the findings. The sample was predominantly Malaysian (99.5%), with small representations from Indonesia and Vietnam (0.3% each). Female participants made up the majority at 71.3%, compared to 28.7% male respondents. Marital status varied, with 52% married, 43.8% unmarried, and the remainder either widowed or divorced. Most participants (76.6%) were aged between 25 and less than 45 years, highlighting a youthful to mid-life demographic. Additionally, many respondents came from middle to upper-income households, with 93.5% reporting a monthly family income between RM5,001 and RM20,000.

In terms of education, over half (55.5%) of the respondents held a bachelor’s degree, and 20% had completed a certificate or diploma, indicating a relatively well-educated sample. Occupational distribution showed a mix of managerial roles (29.8%), administrative or clerical positions (22.8%), businessmen (20.5%), and students (16.5%). The occupational diversity, combined with varied educational backgrounds and income levels, reflects a broad cross-section of e-health and social media users. The sample’s composition enhances the study’s relevance and applicability to understanding digital mental health engagement among the Malaysian population.

### Patterns and behaviors

Nearly all respondents (99.8%) reported daily use of digital media, with only a small fraction (0.3%) accessing it 2–3 times weekly. Most participants used digital platforms during spare moments (51.5%) or in their free time (48%), with minimal use during school, work, or meals. WhatsApp was the most widely used digital mental health platform, with all 400 respondents indicating its use, followed by Facebook (387 users) and Instagram (371 users), highlighting their central role in communication and information sharing.

The motivations behind digital media use were varied: 287 respondents sought to increase knowledge, 197 engaged in reading or sharing reviews, 167 shared personal experiences, and 120 used the platforms to seek advice or support. Other common motivations included emotional expression, involvement in product-related activities, and helping others. These patterns reflect the broad and multifaceted nature of digital media engagement among users, emphasizing its importance in everyday communication and digital mental health support.

### Model fit evaluation

Table 1 presents key model fit indices for the structural equation modeling (SEM) analysis. The Standardized Root Mean Squared Residual (SRMR) value of 0.092 for both the estimated and saturated models indicates a good fit, as SRMR values below 0.08 to 0.10 typically suggest acceptable model fit. The Chi-Square statistics are 9717.94 for both models, though it requires additional context or comparison to degrees of freedom for conclusive interpretation (31). The Normed Fit Index (NFI) is 0.656, which is lower than the typical benchmark of 0.90, suggesting potential for model improvement. The d_ULS and d_G values remain consistent at 14.095 and 5.299, respectively, indicating model stability. Overall, while the fit indices suggest the model is reasonable, adjustments could be made to improve fitness further (32).

**Table 1:** Model fit evaluation.

Model fit summary	Estimated model	Saturated model
Standardised root mean squared residual (SRMR)	0.092	0.092
d_ULS	14.095	14.095
d_G	5.299	5.299
Chi-Square	9717.94	9717.94
NFI	0.656	0.656
rms_theta	N/A	N/A

### Convergent validity and reliability

Table 2 provides a thorough evaluation of the convergent validity and reliability of the reflective measurement models for self-efficacy, self-esteem, perceived social support, and digital mental health engagement. The factors loadings for self-efficacy range from 0.783 to 0.886, indicating significant contributions to the construct. Self-esteem items show even higher loadings (0.889 to 0.916), strongly linking them to the construct. Perceived social support items demonstrate loadings of 0.873 to 0.904, reinforcing their relevance.

**Table 2:** Convergent validity and reliability of reflective measurement models.

Key construct	Items	Loadings	Cronbach’s alpha	Composite reliability	Rho_A	Average variance extracted
Self-efficacy	SE1	0.886	0.960	0.965	0.960	0.736
	SE2	0.886				
	SE3	0.876				
	SE4	0.885				
	SE5	0.846				
	SE6	0.869				
	SE7	0.833				
	SE8	0.852				
	SE9	0.859				
	SE10	0.783				
Self-esteem	S-E1	0.916	0.924	0.946	0.925	0.814
	S-E2	0.900				
	S-E3	0.889				
	S-E4	0.904				
Perceived social support	PSS1	0.897	0.916	0.940	0.916	0.798
	PSS2	0.898				
	PSS3	0.873				
	PSS4	0.904				
Digital mental health engagement	MHP1	0.931	0.770	0.859	0.907	0.680
	MHP2	0.929				
	MHP3	0.555				

Although the factor loading for MHP3 in digital mental health engagement is lower (0.555), other items maintain strong loadings. Cronbach’s alpha and composite reliability values exceed 0.7 for all constructs, ensuring internal consistency. The Rho_A values further affirm reliability, ranging from 0.916 to 0.960. The average variance extracted (AVE) values for self-esteem, perceived social support, and self-efficacy exceed 0.50, confirming convergent validity. The AVE for digital mental health engagement is 0.680, suggesting acceptable validity.

### Direct relationships

Table 3 presents the direct relationships of psychological constructs on digital mental health engagement, showing positive influences from self-efficacy (0.236), self-esteem (0.384), and perceived social support (0.523), with social support being the strongest predictor. Statistical significance is confirmed by *t*-values (4.979, 7.649, 8.384) and *p*-values (*p* = 0.000). High social support enhances e-health engagement, aligning with previous research, while self-efficacy supports health management and self-esteem fosters greater engagement. These findings underscore the importance of strengthening these psychological constructs to improve mental well-being through digital platforms.

**Table 3:** Direct effect of variables.

Main construct	Original sample (O)	T statistics $\left(\left|O/\mathrm{STDEV}\right|\right)$	*p*-value
Self-efficacy-> Digital mental health engagement	0.236	4.979	0.000
Self-esteem-> Digital mental health engagement	0.384	7.649	0.000
Perceived social support-> Digital mental health engagement	0.523	8.384	0.000

### Mediating role of perceived social support

Table 4 highlights the mediating role of perceived social support in the relationships between self-efficacy, self-esteem, and digital mental health engagement. The results show that both self-efficacy and self-esteem directly influence engagement, but these effects are partially mediated by perceived social support. The indirect effect of self-efficacy on engagement through perceived social support is moderate, with a coefficient of 0.123, a statistically significant *t*-statistic of 4.379, and a Variance Accounted For (VAF) value of 49.20%. The effect of self-esteem on engagement through social support is stronger, with a coefficient of 0.201, a *t*-statistic of 5.676, and a VAF value of 32.11%. These findings underscore the importance of enhancing self-efficacy, self-esteem, and social support in digital mental health interventions to improve engagement and effectiveness.

**Table 4:** Mediating effect of the specific indirect effect.

Mediating effect	Original sample (O)	T Statistics $\left(\left|O/\mathrm{STDEV}\right|\right)$	*p*-value	Direct effect	VAF	Type of mediation
Self-efficacy-> Perceived social support-> Digital mental health engagement.	0.123	4.379	0.000	0.127	49.20%	Partial Mediation
Self-esteem-> Perceived social support-> Digital mental health engagement.	0.201	5.676	0.000	0.425	32.11%	Partial mediation

### Acceptance and rejection of research hypotheses

Table 5 presents the results of hypothesis testing, highlighting the acceptance or rejection of each hypothesis based on beta coefficients, *t*-values, and *p*-values. The analysis indicates that all the hypothesized relationships are statistically significant, suggesting that the theoretical framework is robust, and the constructs involved play essential roles in promoting digital mental health engagement.

**Table 5:** Acceptance and rejection of hypotheses.

Hypothesis	Beta coefficient	*t*-value	*p*-value	Significant	Accept/reject
Self-efficacy-> Digital mental health engagement	0.236	4.979	0.000	Significant	Accept
Self-esteem-> Digital mental health engagement	0.384	7.649	0.000	Significant	Accept
Perceived social support-> Digital mental health engagement	0.523	8.384	0.000	Significant	Accept
Self-efficacy-> Perceived social support-> Digital mental health engagement	0.123	4.379	0.000	Significant	Accept
Self-esteem-> Perceived social support-> Digital mental health engagement	0.201	5.676	0.000	Significant	Accept

### Direct relationships of self-efficacy, self-esteem, and perceived social support

The first three hypotheses explore the direct relationships between self-efficacy, self-esteem, perceived social support, and digital mental health engagement. The beta coefficients for these relationships indicate positive correlations, suggesting that higher levels of self-efficacy, self-esteem, and perceived social support contribute to better engagement with digital mental health tools. The relationship between self-efficacy and digital mental health engagement is statistically significant, with a *p*-value less than 0.05 and a *t*-value greater than 1.96. The beta coefficient of 0.236 indicates that increased self-efficacy leads to a moderate improvement in digital mental health engagement, confirming its role in motivating individuals to engage in behaviors that support their digital mental health.

Similarly, the relationship between self-esteem and digital mental health engagement is statistically significant, with a stronger beta coefficient of 0.384 and a *t*-value of 7.649. This suggests that individuals with higher self-esteem are more likely to engage positively with digital mental health tools, indicating that self-esteem plays a significant role in fostering mental well-being. The strongest effect observed is between perceived social support and digital mental health engagement, with a beta coefficient of 0.523 and a *t*-value of 8.384. This highlights that individuals who feel supported by their social networks experience greater digital mental health engagement, underscoring the importance of social relationships and support systems in promoting mental well-being.

### Mediating relationships of perceived social support

The next two hypotheses explore the mediating role of perceived social support in the relationships between self-efficacy and digital mental health engagement, as well as self-esteem and digital mental health engagement. The mediating effect of perceived social support between self-efficacy and digital mental health engagement is significant, with a beta coefficient of 0.123 and a *t*-value of 4.379, indicating that perceived social support partially mediates the relationship. This suggests that while self-efficacy directly influences digital mental health engagement, social support further enhances its effect, with a *p*-value of 0.000 confirming statistical significance. Similarly, the mediating role of perceived social support between self-esteem and digital mental health engagement is significant, with a beta coefficient of 0.201 and a *t*-value of 5.676. The finding shows that individuals with higher self-esteem are more likely to perceive greater social support, which promotes digital mental health engagement. The *p*-value of 0.000 affirms the statistical significance of the mediation, underscoring perceived social support as a key factor in enhancing digital mental health engagement through self-esteem.

### Hypotheses validation

All hypotheses are supported by beta coefficients, *t*-values, and *p*-values, confirming positive relationships between self-efficacy, self-esteem, and perceived social support with digital mental health engagement. Perceived social support mediates these relationships, validating the theoretical framework and emphasizing the need to enhance these constructs in e-health interventions for improved mental well-being.

## Discussion

The study on e-health engagement highlights several significant findings regarding the role of psychological factors in digital mental health engagement. The demographic analysis of 400 e-health users revealed a predominantly Malaysian (99.5%) and female (71.3%) sample, with most respondents falling within the middle to upper-income bracket (93.5%) and possessing a higher education level (55.5% with a bachelor’s degree). High levels of digital media engagement (99.8% using platforms daily) suggest a strong reliance on e-health platforms for knowledge acquisition and social interaction. From a measurement perspective, self-efficacy, self-esteem, and perceived social support exhibited strong validity and reliability, although digital mental health engagement showed slightly lower validity. The structural equation modeling (SEM) analysis revealed that perceived social support exerted the most substantial impact on digital mental health engagement (0.523), followed by self-esteem (0.384) and self-efficacy (0.236), with all relationships demonstrating statistical significance. Notably, the mediating relationships of perceived social support significantly enhanced the influence of self-efficacy and self-esteem on digital mental health engagement, highlighting its essential role in fostering positive digital mental health engagement. These findings emphasize the importance of tailored e-health interventions that strengthen social support mechanisms to enhance engagement and efficacy.

### Strengths and limitations

The research effectively highlights the significant influence of psychological factors on digital mental health engagement, supported by a large, demographically rich Malaysian sample. Strong measurement validity, robust SEM analysis, and statistically significant findings underscore the critical role of perceived social support, offering valuable insights for designing impactful e-health interventions. However, the study’s findings may face limitations in generalizability due to a demographic skew, with a predominance of female, highly educated, and higher-income participants, as digital mental health engagement varies across different population groups (6, 23, 26). Additionally, the study does not thoroughly examine Malaysian sociocultural influences, such as stigma, collectivism, and religious values, which may affect e-health behaviors (16, 17). The use of cross-sectional data restricts causal interpretations (11, 12), while self-reported measures may introduce bias (22).

### Implications

Strengthening social and emotional support networks plays a pivotal role in enhancing digital mental health outcomes. Public health policies should prioritize the integration of digital mental health services within primary care systems and aim to broaden eHealth accessibility, particularly for underserved and marginalized populations (9, 20). In clinical practice, there is a pressing need for clinicians to foster patients’ self-efficacy and reinforce social support structures by encouraging the use of digital mental health platforms and involving family members in therapeutic processes. Interventions that focus on self-esteem, especially cognitive-behavioral therapies, have shown to be effective in managing conditions such as anxiety and depression (9, 16).

From a technological standpoint, the development of eHealth tools must be tailored to meet the needs of at-risk groups through the creation of user-friendly applications that offer both informational and emotional support (1, 15). Additionally, cultural sensitivity must be embedded within digital mental health strategies, as concepts of social support and self-esteem differ significantly across cultural backgrounds. For instance, variations are evident among Mexican American adolescents and individuals living with chronic illnesses in Ghana, emphasizing the importance of culturally aligned digital interventions (2, 23).

### Recommendations

To enhance digital health interventions, it is essential to expand the use of eHealth tools, social media platforms, and mobile applications, particularly for vulnerable populations such as the older adult and those living with chronic illnesses (24, 27). Health systems must prioritize scalability and ensure that these technologies remain accessible, especially for individuals with low digital literacy (1). Equally important is the strengthening of social support systems, which serve as vital buffers against stress and contribute to higher self-esteem (8, 22). Policymakers should work towards creating more connected and supportive environments, especially for individuals recovering from trauma and for aging populations (17, 28).

Promoting self-efficacy should also be a key objective of public health programs. This can be achieved by incorporating strategies that teach problem-solving and resilience-building skills, with particular attention given to marginalized communities (5, 14, 16, 21). Moreover, it is crucial to address the diverse digital mental health needs of different demographic groups. Interventions must be tailored to reflect the unique experiences and requirements of adolescents, individuals with autism, and culturally diverse women (2, 12). Finally, the development of comprehensive digital mental health models should be guided by a holistic perspective. Healthcare policies must integrate psychological constructs such as self-esteem, self-efficacy, and perceived social support, alongside broader societal factors, to effectively support mental well-being (4, 7, 18, 19).

### Future research directions

Future research should examine the evolving roles of social support, self-esteem, and self-efficacy over time, particularly during life transitions, to better understand their impact on digital mental health engagement (19, 26). Investigating how gender, ethnicity, and socioeconomic status intersect with self-esteem and influence engagement is crucial for providing targeted support to vulnerable populations (2, 10). Additionally, evaluating the efficacy of digital mental health interventions in low-resource settings is vital (1, 20). Finally, examining culturally tailored programs is necessary to ensure relevance in diverse contexts (16, 23).

## Conclusion

The study underscores the essential roles of self-efficacy, self-esteem, and perceived social support in driving e-health engagement, with social support serving as a key mediator in these relationships. Emphasizing AI-driven personalization and culturally sensitive strategies, the research advocates for more tailored digital mental health solutions. It integrates psychological constructs into a cohesive framework, demonstrating how their interaction produces a synergistic effect that strengthens user engagement. The multidimensional model offers valuable guidance for developers, healthcare providers, and policymakers in crafting more impactful digital interventions. Ultimately, the findings bridge contextual research gaps and support more inclusive, effective digital mental health engagement across diverse populations.

## Data Availability

The original contributions presented in the study are included in the article/Supplementary material, further inquiries can be directed to the corresponding author.
