# Supplementary material for: Empowering minds: how self-efficacy, self-esteem, and social support drive digital mental health engagement
Source: Front Public Health. 2025 Apr 25;13:1565327. doi: 10.3389/fpubh.2025.1565327 (PMC12061876; doi:10.3389/fpubh.2025.1565327)
Supplement: Supplementary file 1 [file Table_1.pdf]

Appendix A

Exploring the Role of Self-Efficacy, Self-Esteem and Social Support in Digital Mental Health Engagement: A Comparative Analysis of Recent Studies

Reference	Key Focus	Findings	Comparison
Alquaiz et al. (2024)	Violence, social support, self-esteem, and mental health	Explored how violence against women influences mental health, highlighting the mediating role of social support and self-esteem.	Similar to other studies showing social support as a crucial factor for mental health, but with a focus on gender-based violence.
Bandura (1977)	Self-efficacy theory	Proposed self-efficacy as a major factor in behavioral change.	Groundbreaking theory that influences many subsequent studies on self-esteem and health behaviors.
Cohen (2002)	Social relationships and health	Social support positively impacts physical and mental health, mitigating stress and promoting overall well-being.	Often cited in studies on mental health and support networks, linking social relationships to health outcomes.
Evans et al. (2024)	Autistic masking and mental health	Found that autistic masking negatively impacts self-esteem and increases mental health issues.	A unique angle focusing on autism and social support in mental health.
Hazime & Burner (2024)	Social support via ICT for diabetes management	Reviewed how internet communication technology helps in diabetes self-management via social support.	Reflects a growing trend of eHealth's role in chronic disease management, linking to other studies on eHealth's potential.
Jalali et al. (2024)	Pender Health Promotion Model and self-efficacy	Meta-analysis of the impact of health promotion on self-efficacy, showing positive results.	Consistent with Bandura's theories but applied to health promotion.
Jiang et al. (2024)	Media contact, health literacy, and self-efficacy	Found that media contact improves health literacy through perceived social support and self-efficacy.	Highlights the role of media in health behavior, offering insights into digital health interventions.
Khan et al. (2024)	Psychological capital, mental health, and social support	Perceived social support acts as a mediator between psychological capital and mental health in higher education.	Aligns with studies showing the mediating effect of social support on mental health.
Kim (2024)	Personality, self-esteem, and mental health in nursing students	Investigated how self-esteem and personality types affect mental health in nursing students.	Adds to research on self-esteem's role in nursing student mental health, similar to Bandura's work.
Kreski et al. (2024)	Self-esteem and externalizing symptoms in adolescents	Identified correlations between self-esteem and externalizing mental health symptoms over time.	Extends research on the relationship between self-esteem and mental health, particularly in adolescence.
Lai et al. (2024)	eHealth for emotional well-being in cancer families	Found that eHealth improves emotional well-being, mediated by self-efficacy and cancer fatalism.	Unique in its focus on cancer families, adding to the growing literature on eHealth interventions.
Leary & Baumeister (2000)	Sociometer theory of self-esteem	Suggested self-esteem is a sociometer, reflecting an individual's relational value.	A foundational work that influences much of the research on self-esteem and social interactions.
Lin-Lewry et al. (2024)	Digital parenting, self-efficacy, and depression	Digital parenting interventions reduce depressive symptoms by enhancing self-efficacy and social support.	Focuses on parenting interventions, adding a unique perspective to digital health interventions.
Liu et al. (2024)	eHealth literacy and physical activity	Found that eHealth literacy promotes physical activity via health literacy in Chinese college students.	Adds to the understanding of eHealth literacy, particularly in promoting physical activity.
Merino et al. (2024)	Body perceptions, social media, and mental health	Social media's impact on body image is influenced by cultural and gender factors, affecting self-esteem.	Extends the literature on the negative impacts of social media on body image, particularly regarding gender and culture.
Mommaerts et al. (2024)	Anxiety, self-esteem, and mental health in adolescents	Identified anxiety as a key factor in lowering self-esteem in Mexican American adolescents.	Focuses on a specific cultural group, extending the general findings on anxiety and self-esteem.
Nhan et al. (2024)	Online behaviors, mental health, and social network usage	Found that passive social media use is associated with depression through envy and self-esteem issues.	Reflects growing concern over the negative effects of passive social media use on mental health.
Ochnik et al. (2024)	eHealth tools and mental health	Investigated the use of eHealth tools for mental health improvement.	Reinforces the growing literature on the effectiveness of eHealth in mental health interventions.
Pajares (1997)	Self-efficacy research	Overview of self-efficacy theory and its impact on motivation and achievement.	A seminal work foundational to much of the self-efficacy research that follows.
Pitkow & Schueller (2024)	Mental health and eHealth technology	Explores the role of eHealth technologies in improving mental health outcomes.	Aligns with other studies focusing on the role of technology in mental health care.
Rodrigues et al. (2024)	eHealth interventions and user expectations	Examined user expectations of eHealth interventions, highlighting differences in human-supported vs. self-help models.	Adds a user-experience angle to the growing literature on eHealth intervention efficacy.
Rosenberg (1965)	Adolescent self-image and society	Explored how societal factors influence adolescent self-esteem.	Foundational in understanding self-esteem in the context of adolescence.
Suarez et al. (2024)	Online communities and cancer survivors	Identified how online communities offer social support to breast cancer survivors.	Contributes to research on online social support for individuals with health conditions.
Tian (2024)	Social support in digital health services	Explored how digital health services provide social support, enhancing health outcomes.	Reflects the increasing role of digital platforms in providing social support for health.
Uchino (2009)	Social support and physical health	Reviewed how perceived and received social support influences physical health across the lifespan.	Comprehensive and influential work on the health benefits of social support.
Warner & Schwarzer (2024)	Self-efficacy and health	Explored the role of self-efficacy in health behavior and mental health.	A well-established theory that reinforces self-efficacy's critical role in health outcomes.
Wiens et al. (2024)	eHealth for young mothers	Reviewed how eHealth interventions can support mental health in young mothers.	Adds a demographic-specific focus on the potential of eHealth for mental health.
Witts et al. (2024)	Social support and treatment adherence	Found that social support influences treatment adherence among chronic disease patients in Ghana.	Similar findings to studies linking social support with better health outcomes.
Zell & Johansson (2024)	Self-esteem, health, and well-being	Quantitative synthesis of the relationship between self-esteem and health, across 40 meta-analyses.	Provides a robust meta-analysis, aligning with many studies linking self-esteem to health.
Zhu & Li (2024)	Online health information and social support	Found that online health information seeking is influenced by emotional or informational support.	Extends research on online health behaviors, showing how social support affects health information-seeking.
